# Association of Maternal Cigarette Smoking and Smoking Cessation With Preterm Birth

**DOI:** 10.1001/jamanetworkopen.2019.2514

**Published:** 2019-04-19

**Authors:** Samir Soneji, Hiram Beltrán-Sánchez

**Affiliations:** 1 The Dartmouth Institute for Health Policy & Clinical Practice, Dartmouth Geisel School of Medicine, Lebanon, New Hampshire; 2 Dartmouth-Hitchcock Norris Cotton Cancer Center, Dartmouth Geisel School of Medicine, Lebanon, New Hampshire; 3 Department of Community Health Sciences, University of California, Los Angeles; 4 California Center for Population Research, Los Angeles

## Abstract

**Importance:**

Cigarette smoking during pregnancy increases the risk of preterm birth, low birth weight, and infant mortality.

**Objective:**

To assess the probability of preterm birth among expectant mothers who smoked cigarettes before pregnancy and quit smoking at the start or during pregnancy.

**Design, Setting, and Participants:**

This cross-sectional study analyzed information provided on live birth certificates from 2011 through 2017 that were obtained from US states that implemented the 2003 revision of the US live birth certificate. In total, 25 233 503 expectant mothers who delivered live neonates and had known prepregnancy and trimester-specific cigarette smoking frequency were included in the analyses.

**Exposures:**

Cigarette smoking frequency (1-9, 10-19, and ≥20 cigarettes per day) 3 months prior to pregnancy and for each trimester during pregnancy.

**Main Outcomes and Measures:**

Cigarette smoking cessation throughout pregnancy, after the first trimester, after the second trimester, and during the third trimester irrespective of first and second trimester smoking. Probability of preterm birth (<37 weeks’ gestation).

**Results:**

Of 25 233 503 expectant mothers who delivered live neonates between 2011 and 2017, the modal age at delivery was 25 to 29 years; 52.9% were non-Hispanic white, 23.6% were Hispanic, and 14.2% were non-Hispanic black women; 22 600 196 mothers did not smoke during the 3 months prior to pregnancy, and 2 633 307 smoked during the 3 months prior to pregnancy. The proportion of prepregnancy smokers who quit throughout pregnancy was 24.3% in 2011 and 24.6% in 2017. The proportion of prepregnancy smokers who quit during the third trimester was 39.5% in 2011 and 39.7% in 2017. High-frequency cigarette smoking often occurred among expectant mothers who smoked during pregnancy (eg, 46.9% of third-trimester smokers smoked ≥10 cigarettes per day in 2017). The probability of preterm birth decreased more the earlier smoking cessation occurred in pregnancy. For example, the probability of preterm birth was 9.8% (95% CI, 9.7%-10.0%) among 25- to 29-year-old, non-Hispanic white, primigravida and primiparous expectant mothers (ie, pregnant for the first time and not yet delivered) who smoked 1 to 9 cigarettes per day prior to pregnancy and maintained this frequency throughout their pregnancy. The probability of preterm birth was 9.0% (95% CI, 8.8%-9.1%) if smoking cessation occurred at the start of the second trimester (an 8.9% relative decrease), and 7.8% (95% CI, 7.7%-8.0%) if cessation occurred at the start of pregnancy (a 20.3% relative decrease).

**Conclusions and Relevance:**

Quitting smoking—and quitting early in pregnancy—was associated with reduced risk of preterm birth even for high-frequency cigarette smokers.

## Key Points

**Question:**

Does an association exist between maternal cigarette smoking cessation during pregnancy and risk of preterm birth?

**Findings:**

In this cross-sectional study of more than 25 million pregnant women, only approximately 1 of 4 women who smoked prior to pregnancy quit throughout pregnancy, and approximately 1 of 2 women who smoked during their pregnancy smoked 10 or more cigarettes per day. Cigarette smoking cessation, especially early in pregnancy, was associated with reduced risk of preterm birth (relative decrease up to 20%) even for high-frequency cigarette smokers.

**Meaning:**

Greater emphasis on smoking cessation among expectant mothers who smoke may be associated with a lower burden of preterm birth.

## Teaser

This cross-sectional study assesses the association between preterm delivery and smoking cigarettes throughout pregnancy or ceasing smoking at the start of or during pregnancy among more than 25 million expectant mothers who smoked cigarettes 3 months before pregnancy.

## Introduction

The proportion of expectant mothers in the United States who smoke during pregnancy has decreased over time.^1^ Yet, 7.2% of expectant mothers in 2016—nearly 283 000—still smoked during pregnancy.^2^ Cigarette smoking during pregnancy leads to many detrimental child health outcomes, including low birth weight, delayed intrauterine development, preterm birth, infant mortality, and long-term developmental delays.^3,4,5,6,7,8,9,10,11,12,13^

A recent Ohio-based study found that women who smoke before pregnancy and quit either at the start of pregnancy or at the start of the second trimester experience approximately the same rate of preterm birth as their nonsmoking counterparts.^14^ Yet, it is not known if this association occurs nationally or varies by the frequency of prepregnancy smoking. The present study addresses these research gaps by assessing temporal patterns in the rate of smoking cessation at the start of and during pregnancy and in the occurrence of high-frequency cigarette smoking. This study also estimates the reduction in the risk of preterm birth associated with smoking cessation at the start of and during pregnancy.

## Methods

### Data

The study used birth certificate data collected by the US National Center for Health Statistics from 2011 through 2017 as part of the National Vital Statistics System.^9,15^ The 2003 revision of the US Standard Certificate of Live Birth ascertains cigarette smoking frequency 3 months prior to pregnancy and trimester-specific cigarette smoking frequency. In 2011, the District of Columbia and 36 states, which together accounted for 83% of all US births in 2011, had implemented the 2003 revision.^16^ By 2016, all states and the District of Columbia had implemented the 2003 revision.^17^ The present study used data from 25 233 503 expectant mothers who had live births occurring in states that had implemented the 2003 revision and who had prepregnancy and trimester-specific smoking frequency data recorded on the birth certificate^16,17,18,19,20,21,22^ (94.2% of all live births between 2011 and 2017; eTable 1 in the Supplement). This study followed the Strengthening the Reporting of Observational Studies in Epidemiology (STROBE) reporting guideline for cross-sectional studies. The Dartmouth College Committee for the Protection of Human Subjects determined institutional review board review was not required for this present study because the regulatory definition of human subjects research (45 CFR 46.102[f]) did not apply. The data are deidentified and publicly available.

### Outcomes

The first outcome was cigarette smoking cessation throughout pregnancy (ie, no smoking during any trimester) among expectant mothers who smoked 1 or more cigarettes 3 months prior to pregnancy. The second outcome was cigarette smoking cessation after the first trimester (ie, no smoking during the second or third trimester) among expectant mothers who smoked 1 or more cigarettes during the 3 months prior to pregnancy and during the first trimester. The third outcome was cigarette smoking cessation after the second trimester (ie, no smoking during the third trimester) among expectant mothers who smoked 1 or more cigarettes 3 months prior to pregnancy and during the first and second trimesters. The fourth outcome was cigarette smoking cessation during the third trimester irrespective of cigarette smoking during the first or second trimester among expectant mothers who smoked 1 or more cigarettes 3 months prior to pregnancy.^23^ The fifth outcome was preterm birth (ie, delivery at <37 weeks’ gestation).

### Covariates

Sociodemographic characteristics of expectant mothers included age at delivery (<15, 15-19, and so forth to 50-54 years), race/ethnicity (Hispanic, non-Hispanic black, non-Hispanic white, non-Hispanic other race, or unknown race), educational attainment (less than high school graduate, high school graduate, or at least some college), marital status (married or unmarried), receipt of Special Supplemental Nutrition Program for Women, Infants, and Children (WIC) benefits, and source of payment for the delivery (Medicaid, private insurance, self-pay, or other). Pregnancy history included lifetime number of pregnancies (gravida), pregnancies that resulted in birth of viable offspring (para), and pregnancies that did not result in the birth of viable offspring (abortus). Smoking frequency was categorized as 0, 1 to 9, 10 to 19, and 20 or more cigarettes per day 3 months prior to pregnancy and in the first, second, or third trimesters. The proportion of births with missing data on any of these covariates ranged between 0% and 3.0% (eTable 2 in the Supplement).

### Statistical Analysis

First, we assessed the distribution of sociodemographic characteristics and pregnancy history for women with live births in US states that ascertained trimester-specific smoking frequency (ie, states that adopted the 2003 revision of the US live birth certificate). Second, we calculated the annual proportion of expectant mothers who (1) smoked prior to pregnancy but quit throughout pregnancy, (2) smoked prior to pregnancy and during the first trimester but quit during the second and third trimesters, (3) smoked prior to pregnancy and during the first and second trimesters but quit during the third trimester, and (4) who smoked prior to pregnancy but quit during the third trimester, irrespective of first or second trimester smoking. We tested for time trends by fitting a least squares regression line of each proportion against the year. Third, we calculated the proportion of smokers who smoked 1 to 9, 10 to 19, and 20 or more cigarettes per day by trimester over time and similarly tested for time trends.

Fourth, we calculated the proportion of neonates delivered at less than 37 weeks’ gestation (ie, preterm) among expectant mothers who did not smoke, those who smoked during the 3 months prior to pregnancy, and those who smoked during each trimester. We assessed differences between each pair of trimester-specific proportions using the χ^2^ test for equality of proportions.

Fifth, we fit a series of 5 multivariable logistic regression models to ascertain characteristics associated with each outcome. The outcome of model 1 was cessation throughout pregnancy; the covariates included the year of birth, sociodemographic characteristics (age at delivery, race/ethnicity, educational attainment, marital status, receipt of WIC benefits, and source of payment for delivery), pregnancy history (gravida, para, and abortus), and prepregnancy smoking frequency. The outcome of model 2 was cessation after the first trimester and included the same covariates as model 1 as well as first trimester smoking frequency. The outcome of model 3 was cessation after the second trimester and included the same covariates as model 2 as well as second trimester smoking frequency. The outcome of model 4 was cessation during the third trimester irrespective of smoking during the first or second trimester and also included the same covariates as model 3. The outcome of model 5 was preterm birth among prepregnancy smokers and included the same covariates as model 3. Finally, the outcome of model 6 was preterm birth among prepregnancy and pregnancy nonsmokers; the covariates included the year of birth, sociodemographic characteristics, and pregnancy history. Listwise deletion was used to remove cases with missing data. Throughout the analysis, a 2-sided *P* < .05 was considered statistically significant.

To illustrate any association between smoking cessation during pregnancy and preterm birth, we estimated the probability of this outcome based on the results of models 5 and 6.^24^ We considered common subpopulations of expectant mothers: primigravida and primiparous; 25 to 29 years of age; non-Hispanic white, non-Hispanic Black, and Hispanic; married; no receipt of WIC benefits; and private insurance. We varied trimester-specific smoking frequency. All analyses were conducted with R, version 3.5.3 (The Comprehensive R Archive Network).

## Results

### Study Sample

The modal maternal age at delivery was 25 to 29 years. Of 25 233 503 expectant mothers, 52.9% were non-Hispanic white, 23.6% were Hispanic, and 14.2% were non-Hispanic black women (eTable 3 in the Supplement). The number of mothers who did not smoke during the 3 months prior to pregnancy was 22 600 196, and 2 633 307 women smoked during the 3 months prior to pregnancy. Most expectant mothers were high school graduates (25.0%) or had at least some college education (59.7%), were married (59.8%), and did not receive WIC benefits (57.1%). The majority of deliveries were paid by private insurance (48.2%) or Medicaid (43.3%).

### Smoking Cessation

**Figure 1.**
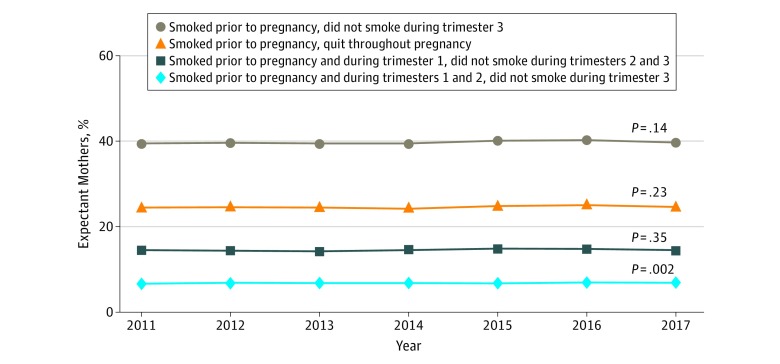
Proportion of Expectant Mothers from 2011 through 2017 Who Smoked 3 Months Prior to Pregnancy but Quit Smoking Throughout Pregnancy, During the Second and Third Trimesters, During the Third Trimester, or in the Third Trimester Analysis of 2011 through 2017 US live birth certificate data.^16,17,18,19,20,21,22^

The proportion of expectant mothers who smoked 3 months prior to pregnancy and quit throughout pregnancy equaled 24.3% in 2011 and 24.6% in 2017 (*P* = .23; Figure 1). The proportion of expectant mothers who smoked 3 months prior to pregnancy and during the first trimester but quit for the remainder of pregnancy equaled 14.5% in 2011 and 14.4% in 2017 (*P* = .35). The proportion of expectant mothers who smoked 3 months prior to pregnancy and during the first and second trimesters but quit for the remainder of pregnancy increased from 6.7% in 2011 to 6.8% in 2017 (*P* = .002). Finally, the proportion of expectant mothers who smoked 3 months prior to pregnancy and quit during the third trimester, irrespective of first or second trimester smoking, equaled 39.5% in 2011 and 39.7% in 2017 (*P* = .14).

### Frequency of Cigarette Smoking

**Figure 2.**
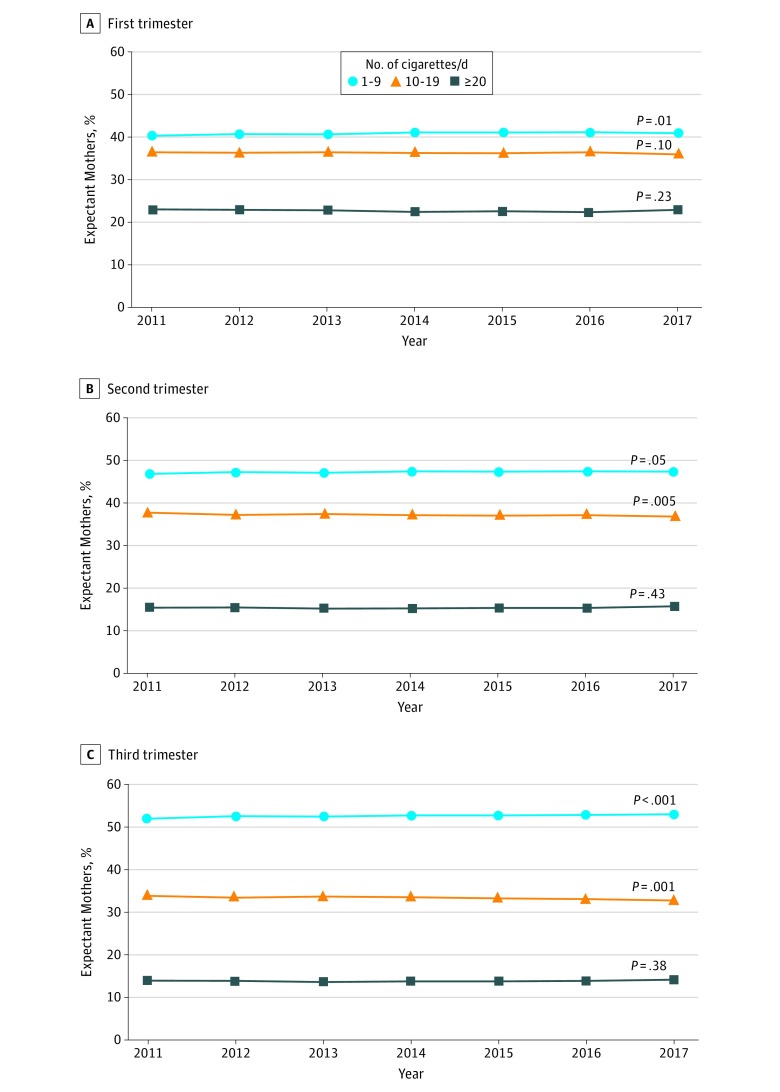
Frequency of Cigarette Smoking by Trimester Among Expectant Mothers Who Smoked During Pregnancy Analysis of 2011 through 2017 US live birth certificate data.^16,17,18,19,20,21,22^

High-frequency smoking commonly occurred during all 3 trimesters among expectant mothers who smoked (Figure 2). For example, 46.9% of third-trimester smokers smoked 10 or more cigarettes per day in 2017. In addition, the proportion of third trimester smokers who smoked 10 to 19 cigarettes per day decreased over time from 33.9% in 2011 to 32.8% in 2017 (*P* = .001), whereas the proportion of third trimester smokers who smoked 20 or more cigarettes per day remained approximately constant over time, from 13.9% in 2011 to 14.2% in 2017 (*P* = .38).

### Preterm Birth

**Figure 3.**
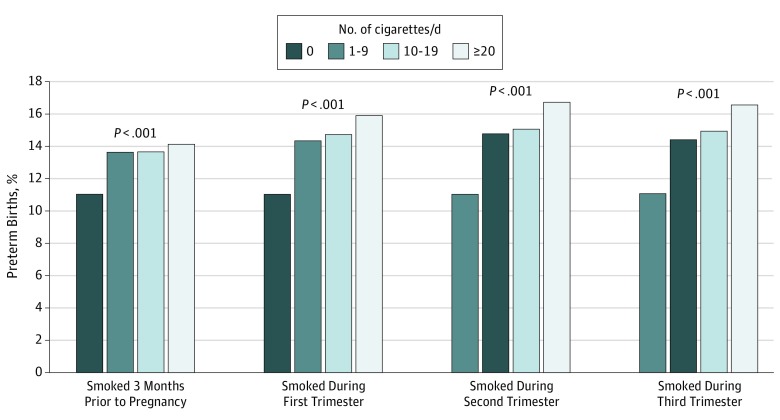
Probability of Preterm Birth (<37 Weeks’ Gestation) by Cigarette Smoking Status 3 Months Prior to Pregnancy or During Trimester 1, 2, or 3 Analysis of 2011 through 2017 US live birth certificate data.^16,17,18,19,20,21,22^

The proportion of preterm births increased with smoking frequency in each trimester (Figure 3). For example, among first trimester smokers, the proportion equaled 14.4% for those who smoked 1 to 9 cigarettes per day, 14.8% for those who smoked 10 to 19 cigarettes per day, and 16.0% for those who smoked 20 or more cigarettes per day. Among third trimester smokers, the probability equaled 14.5% for those who smoked 1 to 9 cigarettes per day, 15.0% for those who smoked 10 to 19 cigarettes per day, and 16.6% for those who smoked 20 or more cigarettes per day.

### Regression Analyses

Univariable regression analyses identified significant associations between sociodemographic characteristics, pregnancy history, and trimester-specific smoking frequency with smoking cessation and preterm birth (eTable 4 in the Supplement). On the basis of results of the multivariable regression models, the estimated odds of cessation throughout pregnancy, after the first trimester, after the second trimester, and during the third trimester were all higher for expectant mothers 24 years of age or younger compared with those 25 to 29 years of age (Table). For example, the adjusted odds ratio (aOR) equaled 1.02 (95% CI, 1.01-1.02) for cessation throughout pregnancy for 20- to 24-year-old expectant mothers who smoked prior to pregnancy compared with those aged 25 to 29 years. Conversely, the odds of cessation at each of these pregnancy time points was lower for expectant mothers aged 30 to 44 years compared with those aged 25 to 29 years. The odds of cessation at each of these pregnancy time points was higher for Hispanic and non-Hispanic black expectant mothers compared with non-Hispanic white expectant mothers (eg, aOR, 1.44; 95% CI, 1.42-1.45 for cessation throughout pregnancy for non-Hispanic black expectant mothers who smoked prior to pregnancy). Receipt of WIC benefits and enrollment in Medicaid insurance were associated with lower odds of cessation at each of these pregnancy time points. Finally, higher gravidity and parity levels were associated with lower odds of cessation at each of these pregnancy time points, too.

**Table.** Multivariable Regression Results for Smoking Cessation at Various Points in Pregnancy and Preterm Birth^a^

Covariate	Adjusted Odds Ratio (95% CI)					
	Cessation Throughout Pregnancy (n = 2 542 018)^b^	Cessation After Trimester 1 (n = 2 538 338)^c^	Cessation After Trimester 2 (n = 1 596 099)^d^	Cessation in Trimester 3 (n = 2 538 338)^c^	Preterm Birth Among Prepregnancy Smokers (n = 2 537 393)^e^	Preterm Birth Among Prepregnancy and Pregnancy Nonsmokers (n = 21 344 623)^f^
Year of delivery (reference, 2011)						
2012	1.02 (1.01-1.03)	0.99 (0.98-1.01)	1.02 (0.99-1.04)	1.02 (1.00-1.04)	0.98 (0.97-1.00)	0.99 (0.98-0.99)
2013	1.02 (1.00-1.03)	0.99 (0.97-1.00)	1.03 (1.01-1.06)	1.03 (1.01-1.06)	0.99 (0.98-1.00)	0.97 (0.96-0.97)
2014	1.01 (1.00-1.02)	1.03 (1.01-1.04)	1.05 (1.02-1.07)	1.06 (1.04-1.08)	1.00 (0.99-1.02)	0.97 (0.96-0.97)
2015	1.05 (1.03-1.06)	1.06 (1.04-1.08)	1.06 (1.03-1.08)	1.07 (1.05-1.10)	1.01 (0.99-1.02)	0.97 (0.96-0.97)
2016	1.07 (1.06-1.08)	1.07 (1.05-1.09)	1.08 (1.05-1.10)	1.09 (1.07-1.11)	1.03 (1.01-1.04)	0.98 (0.97-0.98)
2017	1.05 (1.04-1.06)	1.04 (1.02-1.06)	1.08 (1.05-1.11)	1.08 (1.06-1.11)	1.07 (1.05-1.08)	1.03 (1.03-1.04)
Age at delivery (reference, 25-29), y						
<15	1.22 (1.00-1.49)	2.19 (1.74-2.74)	2.10 (1.49-2.95)	1.79 (1.29-2.47)	1.41 (1.13-1.76)	1.76 (1.70-1.83)
15-19	1.16 (1.15-1.18)	1.36 (1.34-1.38)	1.31 (1.28-1.35)	1.18 (1.16-1.21)	1.00 (0.98-1.01)	1.14 (1.13-1.15)
20-24	1.02 (1.01-1.02)	1.09 (1.08-1.10)	1.13 (1.11-1.15)	1.07 (1.06-1.09)	0.96 (0.95-0.97)	1.02 (1.02-1.03)
30-34	1.01 (1.00-1.02)	0.96 (0.95-0.97)	0.95 (0.93-0.97)	0.98 (0.96-0.99)	1.14 (1.13-1.16)	1.05 (1.05-1.06)
35-39	0.95 (0.94-0.96)	0.93 (0.92-0.95)	0.94 (0.92-0.97)	0.97 (0.95-0.99)	1.38 (1.36-1.40)	1.23 (1.22-1.23)
40-44	0.84 (0.82-0.87)	0.88 (0.85-0.92)	0.96 (0.91-1.02)	0.96 (0.91-1.00)	1.65 (1.61-1.70)	1.51 (1.49-1.52)
45-49	0.81 (0.70-0.94)	0.89 (0.73-1.08)	0.90 (0.67-1.22)	0.96 (0.74-1.23)	1.76 (1.54-2.01)	1.77 (1.73-1.82)
50-54	0.55 (0.24-1.27)	0.99 (0.38-2.56)	0.52 (0.07-3.85)	0.77 (0.19-3.15)	1.60 (0.80-3.22)	2.05 (1.88-2.22)
Race/ethnicity (reference, non-Hispanic white)						
Hispanic	2.35 (2.32-2.38)	2.15 (2.12-2.19)	1.65 (1.61-1.69)	1.70 (1.67-1.74)	1.08 (1.06-1.10)	1.12 (1.12-1.13)
Non-Hispanic black	1.44 (1.42-1.45)	1.51 (1.49-1.53)	1.44 (1.41-1.47)	1.37 (1.35-1.40)	1.53 (1.51-1.54)	1.60 (1.59-1.60)
Non-Hispanic other	1.28 (1.26-1.29)	1.41 (1.39-1.44)	1.34 (1.31-1.38)	1.28 (1.25-1.31)	1.13 (1.11-1.15)	1.11 (1.10-1.11)
Unknown	1.28 (1.20-1.35)	1.06 (0.98-1.16)	1.21 (1.07-1.36)	1.09 (0.98-1.21)	1.19 (1.11-1.27)	1.12 (1.10-1.15)
Educational attainment (reference, <high school)						
High school graduate	1.51 (1.49-1.52)	1.45 (1.43-1.47)	1.26 (1.24-1.28)	1.26 (1.24-1.28)	0.88 (0.87-0.89)	0.93 (0.93-0.94)
At least some college	2.35 (2.33-2.38)	2.13 (2.10-2.16)	1.59 (1.56-1.62)	1.60 (1.57-1.63)	0.82 (0.81-0.83)	0.80 (0.80-0.80)
Marital status (reference, unmarried)	1.48 (1.47-1.49)	1.11 (1.10-1.12)	0.99 (0.98-1.01)	1.03 (1.02-1.05)	0.90 (0.89-0.91)	0.81 (0.80-0.81)
WIC benefits (reference, none)	0.79 (0.79-0.80)	0.87 (0.86-0.88)	0.90 (0.89-0.91)	0.88 (0.87-0.89)	0.80 (0.80-0.81)	0.92 (0.91-0.92)
Source of payment (reference, private insurance)						
Medicaid	0.57 (0.57-0.58)	0.66 (0.66-0.67)	0.86 (0.85-0.88)	0.80 (0.79-0.81)	1.20 (1.19-1.21)	1.13 (1.13-1.13)
Other	0.80 (0.79-0.81)	0.91 (0.89-0.93)	1.09 (1.05-1.13)	1.01 (0.99-1.04)	1.15 (1.13-1.18)	1.10 (1.09-1.10)
Self-pay	0.49 (0.48-0.50)	0.56 (0.54-0.58)	0.84 (0.81-0.88)	0.76 (0.73-0.79)	1.61 (1.57-1.65)	0.96 (0.95-0.97)
Gravida (reference, 1)						
2	0.89 (0.88-0.90)	0.88 (0.86-0.90)	0.95 (0.92-0.98)	0.94 (0.91-0.96)	1.08 (1.06-1.10)	1.06 (1.06-1.07)
3	0.84 (0.82-0.86)	0.84 (0.82-0.87)	0.92 (0.88-0.96)	0.91 (0.88-0.95)	1.14 (1.11-1.16)	1.10 (1.08-1.11)
4	0.79 (0.76-0.82)	0.81 (0.77-0.84)	0.95 (0.89-1.01)	0.93 (0.88-0.98)	1.20 (1.17-1.24)	1.13 (1.12-1.15)
Para (reference, 1)						
2	0.69 (0.68-0.70)	0.68 (0.67-0.69)	0.77 (0.75-0.79)	0.77 (0.75-0.79)	0.90 (0.89-0.92)	0.86 (0.85-0.86)
3	0.55 (0.54-0.57)	0.58 (0.56-0.60)	0.71 (0.68-0.74)	0.69 (0.66-0.72)	0.94 (0.92-0.96)	0.89 (0.88-0.90)
4	0.40 (0.39-0.41)	0.46 (0.45-0.48)	0.63 (0.60-0.66)	0.59 (0.56-0.62)	1.08 (1.05-1.11)	1.00 (0.98-1.01)
Abortus (reference, 0)						
1	1.01 (0.99-1.02)	1.05 (1.03-1.07)	1.05 (1.02-1.07)	1.03 (1.01-1.05)	0.98 (0.97-0.99)	1.01 (1.01-1.02)
2	0.97 (0.95-0.98)	1.04 (1.01-1.06)	1.04 (1.01-1.08)	1.01 (0.98-1.05)	1.01 (0.99-1.02)	1.09 (1.08-1.10)
3	0.91 (0.88-0.93)	1.02 (0.98-1.05)	1.00 (0.95-1.05)	0.98 (0.93-1.02)	1.05 (1.03-1.08)	1.18 (1.17-1.20)
4	0.80 (0.78-0.83)	0.90 (0.87-0.94)	1.01 (0.96-1.06)	0.95 (0.91-1.00)	1.12 (1.09-1.16)	1.30 (1.28-1.31)
Plurality (reference, 1)						
2	NA	NA	NA	NA	10.80 (10.64-10.97)	13.25 (13.18-13.31)
≥3	NA	NA	NA	NA	156.78 (123.24-199.45)	165.78 (157.52-174.46)
Smoking frequency, No. of cigarettes/d (reference, 1-9)						
3 mo prior to pregnancy						
10-19	0.49 (0.49-0.49)	1.30 (1.28-1.31)	1.65 (1.61-1.68)	1.23 (1.21-1.25)	0.95 (0.94-0.96)	NA
≥20	0.31 (0.31-0.32)	1.06 (1.05-1.08)	1.68 (1.64-1.71)	1.11 (1.09-1.12)	0.91 (0.90-0.93)	NA
Trimester 1						
10-19	NA	0.38 (0.37-0.38)	1.12 (1.10-1.15)	NA	NA	NA
≥20	NA	0.39 (0.38-0.39)	1.53 (1.49-1.58)	NA	NA	NA
Trimester 2						
10-19	NA	NA	0.23 (0.23-0.24)	NA	NA	NA
≥20	NA	NA	0.19 (0.18-0.19)	NA	NA	NA
Smoking frequency, No. of cigarettes/d (reference, 0)						
Trimester 1						
1-9	NA	NA		0.41 (0.40-0.42)	1.16 (1.14-1.17)	NA
10-19	NA	NA		0.49 (0.47-0.50)	1.24 (1.22-1.26)	NA
≥20	NA	NA		0.62 (0.60-0.64)	1.30 (1.28-1.33)	NA
Trimester 2						
1-9	NA	NA		0.01 (0.01-0.01)	1.42 (1.39-1.44)	NA
10-19	NA	NA		0.00 (0.00-0.00)	1.50 (1.46-1.53)	NA
≥20	NA	NA		0.00 (0.00-0.00)	1.58 (1.53-1.63)	NA
Trimester 3						
1-9	NA	NA		NA	0.78 (0.77-0.80)	NA
10-19	NA	NA		NA	0.78 (0.76-0.80)	NA
≥20	NA	NA		NA	0.79 (0.77-0.81)	NA

Abbreviation: NA, not applicable; WIC, Special Supplemental Nutrition Program for Women, Infants, and Children.

^a^ Analysis of 2011 through 2017 US live birth certificate data.^16,17,18,19,20,21,22^

^b^ Total of 91 289 observations deleted from regression because missing value on at least 1 covariate (3.5%).

^c^ Total of 94 969 observations deleted from regression because missing value on at least 1 covariate (3.6%).

^d^ Total of 53 801 observations deleted from regression because missing value on at least 1 covariate (3.3%).

^e^ Total of 95 914 observations deleted from regression because missing value on at least 1 covariate (3.6%).

^f^ Total of 1 218 326 observations deleted from regression because missing value on at least 1 covariate (5.4%).

Among expectant mothers who smoked prior to pregnancy, the odds of preterm birth were higher for those who were younger than 15 years of age or were 30 to 49 years of age compared with those 25 to 29 years of age. The odds of preterm birth were also higher for non-Hispanic black (aOR, 1.53; 95% CI, 1.51-1.54) and Hispanic (aOR, 1.08; 95% CI, 1.06-1.10) expectant mothers compared with non-Hispanic white expectant mothers. Receipt of WIC benefits was associated with lower odds of preterm birth (aOR, 0.80; 95% CI, 0.80-0.81).

Increased frequency of cigarette smoking during the first and second trimesters was associated with increased odds of preterm birth. For example, among first trimester smokers, the odds were 1.16 times higher (95% CI, 1.14-1.17) for those who smoked 1 to 9 cigarettes per day, 1.24 times higher (95% CI, 1.22-1.26) for those who smoked 10 to 19 cigarettes per day, and 1.30 times higher (95% CI, 1.28-1.33) for those who smoked 20 or more cigarettes per day compared with those who smoked 0 cigarettes per day in the first trimester (ie, stopped smoking). Among second trimester smokers, the odds of preterm birth were 1.42 times higher (95% CI, 1.39-1.44) for those who smoked 1 to 9 cigarettes per day, 1.50 times higher (95% CI, 1.46-1.53) for those who smoked 10 to 19 cigarettes per day, and 1.58 times higher (95% CI, 1.53-1.63) for those who smoked 20 or more cigarettes per day compared with those who smoked 0 cigarettes per day in the second trimester (ie, stopped smoking).

The same sociodemographic characteristics were associated with the odds of preterm birth among expectant mothers who smoked prior to and during pregnancy as those who did not smoke prior to or during pregnancy. For example, the odds of preterm birth were higher for non-Hispanic black (aOR, 1.60; 95% CI, 1.59-1.60) and Hispanic (aOR, 1.12; 95% CI, 1.12-1.13) nonsmoking expectant mothers compared with non-Hispanic white expectant mothers.

**Figure 4.**
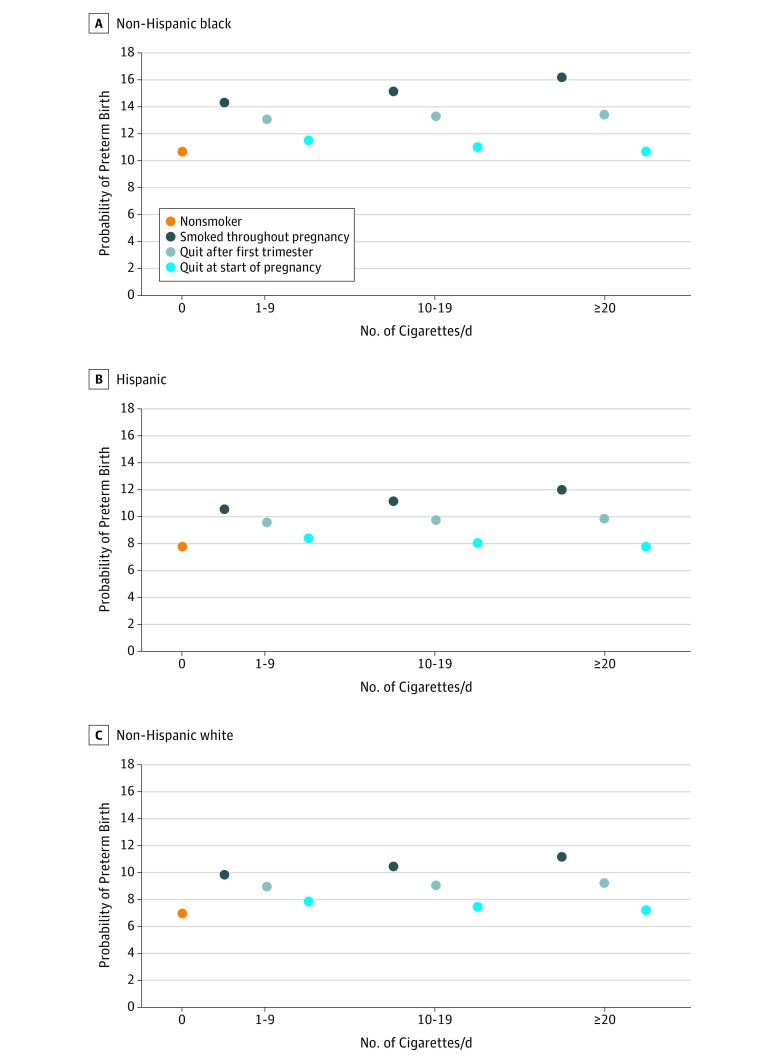
Estimated Probability of Preterm Birth (<37 Weeks’ Gestation) by Cigarette Smoking Frequency Among Expectant Mothers Who Smoked 3 Months Prior to Pregnancy but Quit Smoking at Various Points During Pregnancy and Among Expectant Mothers Who Did Not Smoke 3 Months Prior to or During Pregnancy Analysis of 2011 through 2017 US live birth certificate data.^16,17,18,19,20,21,22^ Expectant primigravida and primiparous women were aged 25 to 29 years, gave birth in 2017, attended at least some college, were married, did not receive Special Supplemental Nutrition Program for Women, Infants, and Children benefits, and were privately insured. The width of the 95% confidence intervals are smaller than the diameters of the circles denoting the point estimates and thus do not show in the figure.

Based on the multivariable regression models, the estimated probability of preterm birth decreased the earlier smoking cessation occurred in pregnancy (Figure 4). For example, the probability of preterm birth equaled 9.8% (95% CI, 9.7%-10.0%) among 25- to 29-year-old, non-Hispanic white, primigravida and primiparous expectant mothers who smoked 1 to 9 cigarettes per day prior to and during their pregnancy. The probability of preterm birth decreased to 9.0% (95% CI, 8.8%-9.1%) if they quit smoking after the first trimester. The probability of preterm birth further decreased to 7.8% (95% CI, 7.7%-8.0%) if they quit at the start of pregnancy (a 23% relative decrease). We estimated a similar association between the probability of preterm birth and time of smoking cessation in pregnancy for non-Hispanic black and Hispanic expectant mothers although the absolute levels were higher. For example, the probability of preterm birth equaled 11.5% (95% CI, 11.3%-11.7%) for non-Hispanic black expectant mothers who smoked 1 to 9 cigarettes per day prior to pregnancy and quit throughout pregnancy.

Notably, the probability of preterm birth was higher for non-Hispanic black expectant mothers who did not smoke prior to or during pregnancy (10.6%; 95% CI, 10.6%-10.7%) than for non-Hispanic white expectant mothers who smoked 1 to 9 cigarettes per day prior to and throughout pregnancy. The probability of preterm birth was approximately equal between non-Hispanic black expectant mothers who did not smoke prior to or during pregnancy and non-Hispanic white expectant mothers who smoked 10 to 19 cigarettes per day prior to and throughout pregnancy (10.5%; 95% CI, 10.3%-10.6%).

## Discussion

Three central findings emerged from this national study of cigarette smoking during pregnancy. First, only approximately 1 of 4 expectant mothers who smoked prior to pregnancy quit smoking throughout pregnancy. Second, about 1 of 2 expectant mothers who smoked during their pregnancy smoked 10 or more cigarettes per day. Third, smoking cessation—especially early in pregnancy—was associated with reduced risk of preterm birth.

Our study contributes to a growing literature on cigarette smoking during pregnancy.^25,26^ Data from the US Centers for Disease Control and Prevention Pregnancy Risk Assessment Monitoring System found that the proportion of women who smoked 3 months prior to pregnancy and quit before the start of the third trimester equaled 43.2% in 2000 and 54.3% in 2010.^23,27^ However, our study found that this proportion remained essentially stable at 40% between 2011 and 2017. Moore et al^14^ found Ohioan women who quit smoking either at the start of pregnancy or after the first trimester experienced about the same probability of preterm birth as nonsmoking women. By contrast, our national study found a dose-response–type association among women who smoked before pregnancy: the risk of preterm birth was highest among those who smoked throughout pregnancy, lower for those who quit after the first trimester, and still lower for those who quit throughout pregnancy. However, the risk of preterm birth was still higher for prepregnancy smokers who quit throughout pregnancy than for prepregnancy nonsmokers.

Our study found that the odds of preterm birth were lower—not higher—for expectant mothers who smoked prior to pregnancy and in the third trimester compared with those who smoked prior to pregnancy but not in the third trimester. This association may occur for many of the same potential explanations of the birth-weight paradox: infant mortality is lower among low-birth-weight neonates born to smokers than among low-birth-weight neonates born to nonsmokers.^28,29^ First, by not measuring other causes of preterm birth, such as malnutrition and lead exposure, our study may introduce a confounding bias.^30,31,32^ Second, fetuses of prepregnancy smokers who smoked in the third trimester may be different from fetuses of prepregnancy smokers who did not smoke in the third trimester because of higher risk of spontaneous abortion in the former.^33^ A third potential explanation—third trimester smoking is protective against preterm birth—is likely not biologically plausible because third trimester smoking is associated with increased risk of fetal growth restriction.^34^

Our study contributes to a well-established body of evidence on racial and ethnic disparities in the risk of preterm birth by identifying these disparities among both nonsmokers and smokers.^35^ In a regional-based study conducted between 1995 and 2001, Holzman et al^36^ found that black nonsmokers experienced higher rates of preterm delivery than both white nonsmokers and smokers. Our national study similarly found these respective rates have continued in recent years: non-Hispanic black women who did not smoke either prior to or during pregnancy experienced a higher probability of preterm birth than non-Hispanic white women who smoked both prior to and throughout pregnancy.

Expectant mothers who smoke may face greater challenges in quitting compared with their counterparts who are not pregnant. First, the clearance of nicotine is approximately 60% higher among pregnant smokers compared with nonpregnant smokers.^37^ Thus, the physiological experience of nicotine withdrawal comes sooner for the former compared with the latter.^38^ Second, fewer modalities of smoking cessation may be available to pregnant smokers. Among nonpregnant adult cigarette smokers, behavioral and pharmacotherapy interventions (including nicotine replacement therapy) efficaciously and effectively increase the rate of smoking cessation.^39^ However, for intervention among pregnant women who smoke, the US Preventive Services Task Force recommends only behavioral modifications, concluding that the evidence on pharmacotherapy for smoking cessation is insufficient and that the evidence on nicotine replacement therapy is limited and conflicting.^40,41,42^ On the basis of the paucity of information on the effectiveness and safety of nicotine replacement therapy use during pregnancy, the US Food and Drug Administration classifies varenicline, for example, as a category C drug.^43^ Thus, some health care professionals may not prescribe the drug to their pregnant patients who smoke.

### Limitations

We note several limitations of the present analysis. First, publicly available birth certificate data has not identified the state in which the birth occurred since 2005. Thus, we are unable to associate state-specific characteristics of cigarette smoking (eg, mean price per pack of cigarettes) with the occurrence of smoking cessation during pregnancy. Second, states adopted the 2003 revision of the US live birth certificate, which ascertains trimester-specific smoking frequency, in different years. Our temporal analysis—based on this revision—included 83% of all live births in 2011, 90% of all live births in 2014, and 100% of all live births in 2016 and 2017. The estimates of cessation may be biased if smoking patterns differ between states that had and those that had not implemented the 2003 revision of the live birth certificate in a given calendar year. However, the prevalence of current cigarette smoking among reproductive aged women, the proportion of every day smokers among female current cigarette smokers, and the proportion of female current cigarette smokers who attempted to quit in the past year were approximately equal between states that had and those that had not adopted the 2003 revision of the US live birth certificate (eFigure in the Supplement). Second, smoking cessation was ascertained on the basis of self-reported smoking frequency, not cotinine-based confirmation. Expectant mothers may underreport actual smoking frequency and therefore overreport smoking cessation if they perceive smoking during pregnancy as deviant behavior.^44,45,46^ However, a previous study validating maternal self-reported smoking on birth certificates with cotinine levels in newborns found that 85% of mothers who self-reported as nonsmokers were indeed nonsmokers.^47^ Third, birth certificate data do not ascertain smoking after pregnancy. Approximately half of new mothers who quit smoking during pregnancy relapse within 6 months of delivery; relapse may negatively affect infant health and fetal development for future pregnancies.^27^ Fourth, birth certificate data do not report alcohol use during pregnancy; high frequency of alcohol consumption may be associated with an increased risk of preterm birth.^48^ Fifth, birth certificate data do not report paternal smoking during pregnancy; exposure to secondhand tobacco smoke may also be associated with an increased risk of preterm birth.^49^

## Conclusions

In conclusion, cigarette smoking continues to represent a public health burden for women during pregnancy. Cigarette smoking cessation may be especially difficult for pregnant women. However, quitting—and quitting early in pregnancy—was associated with reduced risk of preterm birth even for high-frequency cigarette smokers.

## Notes

Supplement. **eTable 1.** Number of Births **eTable 2.** Missing Data by Covariate **eTable 3.** Description of Sample of Births Occurring in States That Adopted 2003 Revision of US Live Birth Certificate **eTable 4.** Univariable Regression Results for Smoking Cessation at Various Points in Pregnancy and Premature Birth **eFigure.** Prevalence of Current Cigarette Smoking Among Females Aged 18-44 Years, Every Day Smoking Among Current Cigarette Smokers, and Quit Attempt Within Past Year Among Female Current Cigarette Smokers
