# Supplementary material for: Association of Maternal Cigarette Smoking and Smoking Cessation With Preterm Birth
Source: JAMA Netw Open. 2019 Apr 19;2(4):e192514. doi: 10.1001/jamanetworkopen.2019.2514 (PMC6481448; doi:10.1001/jamanetworkopen.2019.2514)
Supplement: Supplement. — eTable 1. Number of Births eTable 2. Missing Data by Covariate eTable 3. Description of Sample of Births Occurring in States That Adopted 2003 Revision of US Live Birth Certificate eTable 4. Univariable Regression Results for Smoking Cessation at Various Points in Pregnancy and Premature Birth eFigure. Prevalence of Current Cigarette Smoking Among Females Aged 18-44 Years, Every Day Smoking Among Current Cigarette Smokers, and Quit Attempt Within Past Year Among Female Current Cigarette Smokers [file jamanetwopen-2-e192514-s001.pdf]

Supplementary Online Content

Soneji S, Beltrán-Sánchez H. Association of maternal cigarette smoking and smoking cessation with preterm birth. *JAMA Netw Open*. 2019;2(4):e192514. doi:10.1001/jamanetworkopen.2019.2514

eTable 1. Number of Births

eTable 2. Missing Data by Covariate

eTable 3. Description of Sample of Births Occurring in States That Adopted 2003 Revision of US Live Birth Certificate

eTable 4. Univariable Regression Results for Smoking Cessation at Various Points in Pregnancy and Premature Birth

eFigure. Prevalence of Current Cigarette Smoking Among Females Aged 18-44 Years, Every Day Smoking Among Current Cigarette Smokers, and Quit Attempt Within Past Year Among Female Current Cigarette Smokers

This supplementary material has been provided by the authors to give readers additional information about their work.

© 2019 Soneji S et al. *JAMA Network Open*.

eTable 1. Number of Births

Year of Delivery	Total Births	States That Adopted 2003 Revision of U.S. Live Birth Certificate	Births Occurring in States that Did Not Adopt 2003 Revision of U.S. Live Birth Certificate	Births Occurring in States that Adopted 2003 Revision of U.S. Live Birth Certificate		
				Total	Missing Cigarette Smoking Frequency on Birth Certificates	Reported Cigarette Smoking Frequency on Birth Certificates
2011	3,953,590	36 & DC[16]	561,726 (14.2%)	3,391,864 (85.8%)	262,720 (7.7%)	3,129,144 (92.3%)
2012	3,952,841	38 & DC[45]	464,054 (11.7%)	3,488,787 (88.3%)	176,892 (5.1%)	3,311,895 (94.9%)
2013	3,932,181	41 & DC[46]	375,149 (9.5%)	3,557,032 (90.5%)	171,111 (4.8%)	3,385,921 (95.2%)
2014	3,988,076	47 & DC[47]	142,788 (3.6%)	3,845,288 (96.4%)	75,122 (2.0%)	3,770,166 (98.0%)
2015	3,978,497	48 & DC[48]	69,013 (1.7%)	3,909,484 (98.3%)	36,218 (0.9%)	3,873,266 (99.1%)
2016	3,945,875	50 & DC[17]	0 (0.0%)	3,945,875 (100.0%)	19,244 (0.5%)	3,926,631 (99.5%)
2017	3,855,500	50 & DC[49]	0 (0.0%)	3,855,500 (100.0%)	19,020 (0.5%)	3,836,480 (99.5%)
Total	27,606,560	—	1,612,730 (5.8%)	25,993,830 (94.2%)	760,327 (2.9%)	25,233,503 (97.1%)

Source: 2011-2017 U.S. Live Birth Certificate data and Births: Final Data for 2011-2017.[16,17,45–49]

© 2019 Soneji S et al. *JAMA Network Open*.

eTable 2. Missing Data by Covariate

Covariate	Proportion Missing
Age at Delivery	0.0%
Race/Ethnicity	0.0%
Educational Attainment	1.2%
Marital Status	1.8%
Receipt of WIC Benefits	1.9%
Source of Payment	1.0%
Gravidity	0.0%
Para	0.0%
Abortus	0.0%
Plurality	0.0%
Smoking Frequency, Three Months Prior to Pregnancy	2.9%
Smoking Frequency, 1st Trimester	2.9%
Smoking Frequency, 2nd Trimester	3.0%
Smoking Frequency, 3rd Trimester	3.0%

© 2019 Soneji S et al. *JAMA Network Open*.

eTable 3. Description of Sample of Births Occurring in States that Adopted 2003 Revision of U.S. Live Birth Certificate

		Trimester-Specific Smoking Frequency on Birth Certificate	
Characteristic	Total	Missing	Recorded
Age at Delivery (Yrs)			
<15	18,791	722 (3.8%)	18,069 (96.2%)
15-19	1,672,406	60,028 (3.6%)	1,612,378 (96.4%)
20-24	5,676,443	186,964 (3.3%)	5,489,479 (96.7%)
25-29	7,494,945	218,753 (2.9%)	7,276,192 (97.1%)
30-34	6,990,497	188,073 (2.7%)	6,802,424 (97.3%)
35-39	3,353,939	84,793 (2.5%)	3,269,146 (97.5%)
40-44	730,922	19,557 (2.7%)	711,365 (97.3%)
45-49	51,142	1,337 (2.6%)	49,805 (97.4%)
50-54	4,745	100 (2.1%)	4,645 (97.9%)
Race/Ethnicity			
Non-Hispanic White	13,786,690	428,545 (3.1%)	13,358,145 (96.9%)
Hispanic	6,055,416	90,052 (1.5%)	5,965,364 (98.5%)
Non-Hispanic Black	3,749,407	174,993 (4.7%)	3,574,414 (95.3%)
Non-Hispanic Other	2,189,216	50,885 (2.3%)	2,138,331 (97.7%)
Unknown	213,101	15,852 (7.4%)	197,249 (92.6%)
Educational Attainment			
Less Than High School	3,930,623	119,586 (3.0%)	3,811,037 (97.0%)
High School Graduate	6,454,117	210,311 (3.3%)	6,243,806 (96.7%)
At Least Some College	15,289,329	404,960 (2.6%)	14,884,369 (97.4%)
Marital Status			
Unmarried	10,289,199	343,859 (3.3%)	9,945,340 (96.7%)
Married	15,233,025	413,885 (2.7%)	14,819,140 (97.3%)
Receipt of WIC Benefits			
No	14,527,895	330,328 (2.3%)	14,197,567 (97.7%)
Yes	10,961,653	313,389 (2.9%)	10,648,264 (97.1%)
Source of Payment			
Private	12,388,915	339,891 (2.7%)	12,049,024 (97.3%)

© 2019 Soneji S et al. *JAMA Network Open*.

Medicaid	11,148,261	333,942 (3.0%)	10,814,319 (97.0%)
Other	1,135,677	37,817 (3.3%)	1,097,860 (96.7%)
Self-Pay	1,057,429	25,108 (2.4%)	1,032,321 (97.6%)
Gravidity			
1	8,281,936	239,658 (2.9%)	8,042,278 (97.1%)
2	7,294,997	200,408 (2.7%)	7,094,589 (97.3%)
3	4,743,885	127,460 (2.7%)	4,616,425 (97.3%)
≥4	5,673,012	192,801 (3.4%)	5,480,211 (96.6%)
Para			
1	10,082,409	288,495 (2.9%)	9,793,914 (97.1%)
2	8,245,060	229,195 (2.8%)	8,015,865 (97.2%)
3	4,360,675	124,630 (2.9%)	4,236,045 (97.1%)
≥4	3,305,686	118,007 (3.6%)	3,187,679 (96.4%)
Abortus			
0	19,349,258	578,357 (3.0%)	18,770,901 (97.0%)
1	4,340,141	102,450 (2.4%)	4,237,691 (97.6%)
2	1,437,801	35,691 (2.5%)	1,402,110 (97.5%)
3	489,652	13,055 (2.7%)	476,597 (97.3%)
≥4	376,978	30,774 (8.2%)	346,204 (91.8%)
Plurality of Current Birth			
1	25,098,043	732,011 (2.9%)	24,366,032 (97.1%)
2	866,243	27,323 (3.2%)	838,920 (96.8%)
≥3	29,544	993 (3.4%)	28,551 (96.6%)

Note: Yrs=Years.

Source: 2011-2017 U.S. Live Birth Certificate data.

© 2019 Soneji S et al. *JAMA Network Open*.

**eTable 4.** Univariable Regression Results for Smoking Cessation at Various Points in Pregnancy and Premature Birth						
	Cessation Throughout Pregnancy (N=2,542,018)	Cessation After 1st Trimester (N=2,538,338)	Cessation After 2nd Trimester (N=1,596,099)	Cessation in 3rd Trimester (N=2,538,338)	Premature Birth Among Pre-Pregnancy Smokers (N=2,537,393)	Premature Birth Among Pre-Pregnancy & Pregnancy Non-Smokers (N=21,344,623)
Covariate	OR (95% CI)	OR (95% CI)	OR (95% CI)	OR (95% CI)	OR (95% CI)	OR (95% CI)
Year of Delivery (Ref: 2011)						
2012	1.01 (1.00, 1.03)	0.99 (0.98, 1.01)	1.01 (0.99, 1.04)	1.01 (1.00, 1.02)	1.00 (0.98, 1.01)	0.98 (0.98, 0.99)
2013	1.01 (1.00, 1.02)	0.97 (0.96, 0.99)	1.01 (0.98, 1.03)	1.00 (0.99, 1.01)	1.01 (1.00, 1.02)	0.97 (0.97, 0.98)
2014	0.99 (0.98, 1.00)	1.00 (0.99, 1.02)	1.01 (0.99, 1.04)	1.00 (0.99, 1.01)	1.03 (1.02, 1.05)	0.97 (0.97, 0.98)
2015	1.02 (1.01, 1.04)	1.02 (1.01, 1.04)	1.02 (0.99, 1.04)	1.03 (1.02, 1.04)	1.04 (1.03, 1.06)	0.97 (0.96, 0.97)
2016	1.04 (1.03, 1.05)	1.02 (1.01, 1.04)	1.03 (1.00, 1.05)	1.04 (1.03, 1.05)	1.08 (1.07, 1.10)	0.98 (0.97, 0.98)
2017	1.01 (1.00, 1.02)	0.99 (0.98, 1.01)	1.03 (1.00, 1.05)	1.01 (1.00, 1.02)	1.14 (1.12, 1.15)	1.00 (0.99, 1.00)
Age at Delivery (Ref: 25-29 Yrs)						
<15	1.09 (0.91, 1.32)	2.26 (1.82, 2.80)	2.28 (1.64, 3.17)	1.74 (1.48, 2.05)	1.31 (1.06, 1.63)	2.34 (2.25, 2.42)
15-19	1.10 (1.09, 1.11)	1.51 (1.49, 1.53)	1.48 (1.44, 1.51)	1.32 (1.31, 1.34)	0.89 (0.88, 0.90)	1.36 (1.36, 1.37)
20-24	1.01 (1.00, 1.01)	1.17 (1.15, 1.18)	1.20 (1.18, 1.22)	1.09 (1.09, 1.10)	0.90 (0.89, 0.90)	1.12 (1.11, 1.12)
30-34	1.10 (1.09, 1.11)	0.98 (0.97, 0.99)	0.95 (0.93, 0.96)	1.05 (1.04, 1.05)	1.17 (1.16, 1.19)	1.04 (1.03, 1.04)
35-39	1.04 (1.03, 1.05)	0.96 (0.94, 0.97)	0.94 (0.91, 0.96)	1.00 (0.99, 1.01)	1.44 (1.43, 1.46)	1.26 (1.26, 1.27)
40-44	0.88 (0.86, 0.91)	0.88 (0.84, 0.91)	0.93 (0.88, 0.99)	0.87 (0.86, 0.89)	1.69 (1.65, 1.74)	1.64 (1.62, 1.65)
45-49	0.82 (0.72, 0.94)	0.85 (0.71, 1.02)	0.81 (0.61, 1.08)	0.81 (0.73, 0.91)	1.91 (1.68, 2.16)	2.82 (2.76, 2.88)

© 2019 Soneji S et al. *JAMA Network Open*.

50-54	0.38 (0.17, 0.82)	0.59 (0.24, 1.48)	0.28 (0.04, 2.00)	0.39 (0.22, 0.72)	1.87 (1.05, 3.33)	4.58 (4.31, 4.87)
Race/Ethnicity (Ref: Non-Hispanic White)						
Hispanic	2.19 (2.16, 2.21)	2.06 (2.03, 2.09)	1.67 (1.63, 1.71)	2.37 (2.35, 2.39)	1.06 (1.05, 1.08)	1.19 (1.18, 1.19)
Non-Hispanic Black	1.14 (1.13, 1.15)	1.33 (1.31, 1.35)	1.38 (1.36, 1.41)	1.27 (1.26, 1.28)	1.67 (1.65, 1.69)	1.80 (1.79, 1.81)
Non-Hispanic Other	1.27 (1.25, 1.29)	1.42 (1.40, 1.45)	1.39 (1.35, 1.43)	1.40 (1.38, 1.41)	1.14 (1.12, 1.16)	1.06 (1.06, 1.07)
Unknown	1.03 (0.99, 1.08)	0.97 (0.91, 1.04)	1.02 (0.92, 1.13)	1.02 (0.98, 1.06)	1.40 (1.33, 1.47)	1.27 (1.26, 1.29)
Educational Attainment (Ref: Less than High School)						
High School Graduate	1.63 (1.62, 1.65)	1.48 (1.46, 1.49)	1.27 (1.25, 1.29)	1.58 (1.57, 1.60)	0.83 (0.82, 0.83)	0.91 (0.91, 0.92)
At Least Some College	3.07 (3.05, 3.10)	2.28 (2.26, 2.31)	1.63 (1.61, 1.66)	2.89 (2.86, 2.91)	0.77 (0.76, 0.77)	0.75 (0.74, 0.75)
Marital Status (Ref: Unmarried)	1.67 (1.66, 1.68)	1.09 (1.08, 1.10)	0.92 (0.91, 0.93)	1.41 (1.40, 1.42)	0.87 (0.86, 0.88)	0.73 (0.73, 0.73)
WIC Benefits (Ref: No)	0.57 (0.57, 0.58)	0.76 (0.76, 0.77)	0.88 (0.87, 0.89)	0.63 (0.62, 0.63)	0.91 (0.90, 0.92)	1.17 (1.17, 1.18)
Source of Payment (Ref: Private Insurance)						
Medicaid	0.37 (0.37, 0.37)	0.50 (0.50, 0.51)	0.72 (0.71, 0.73)	0.39 (0.39, 0.39)	1.30 (1.29, 1.31)	1.29 (1.28, 1.29)
Other	0.65 (0.64, 0.66)	0.77 (0.75, 0.78)	0.96 (0.93, 1.00)	0.68 (0.67, 0.69)	1.19 (1.17, 1.21)	1.12 (1.11, 1.12)
Self-Pay	0.36 (0.35, 0.37)	0.43 (0.41, 0.44)	0.67 (0.64, 0.70)	0.36 (0.35, 0.37)	1.85 (1.81, 1.89)	1.01 (1.00, 1.02)
Gravida (Ref: 1)						
2	0.69 (0.68, 0.69)	0.64 (0.63, 0.64)	0.72 (0.70, 0.73)	0.63 (0.62, 0.63)	1.12 (1.11, 1.13)	0.99 (0.99, 1.00)
3	0.53 (0.52, 0.53)	0.51 (0.50, 0.51)	0.61 (0.60, 0.62)	0.48 (0.47, 0.48)	1.29 (1.27, 1.30)	1.10 (1.10, 1.11)
4	0.36 (0.36, 0.37)	0.39 (0.38, 0.39)	0.54 (0.53, 0.55)	0.34 (0.34, 0.35)	1.75 (1.73, 1.76)	1.39 (1.39, 1.40)
Para (Ref: 1)						

© 2019 Soneji S et al. *JAMA Network Open*.

2	0.61 (0.61, 0.62)	0.57 (0.57, 0.58)	0.66 (0.65, 0.67)	0.56 (0.55, 0.56)	1.12 (1.11, 1.13)	0.97 (0.97, 0.97)
3	0.44 (0.44, 0.44)	0.44 (0.43, 0.44)	0.56 (0.55, 0.57)	0.40 (0.40, 0.41)	1.37 (1.36, 1.38)	1.13 (1.12, 1.13)
4	0.30 (0.30, 0.30)	0.33 (0.32, 0.33)	0.48 (0.47, 0.49)	0.29 (0.28, 0.29)	1.93 (1.91, 1.95)	1.43 (1.43, 1.44)
Abortus (Ref: 0)						
1	0.86 (0.86, 0.87)	0.90 (0.89, 0.90)	0.96 (0.95, 0.98)	0.87 (0.87, 0.88)	1.09 (1.08, 1.10)	1.08 (1.08, 1.08)
2	0.76 (0.75, 0.77)	0.82 (0.80, 0.83)	0.92 (0.90, 0.94)	0.77 (0.77, 0.78)	1.23 (1.21, 1.24)	1.24 (1.24, 1.25)
3	0.67 (0.66, 0.68)	0.76 (0.74, 0.77)	0.87 (0.84, 0.90)	0.69 (0.68, 0.70)	1.34 (1.31, 1.36)	1.41 (1.39, 1.42)
4	0.58 (0.57, 0.59)	0.67 (0.65, 0.69)	0.86 (0.83, 0.90)	0.61 (0.60, 0.62)	1.39 (1.36, 1.42)	1.54 (1.53, 1.56)
Plurality (Ref: 1)						
2	—	—	—	—	10.73 (10.57, 10.89)	12.52 (12.46, 12.58)
≥3	—	—	—	—	139.61 (110.53, 176.33)	146.85 (139.74, 154.33)
Cigarette Smoking Frequency, Three Months Prior to Pregnancy (Ref: 1-9 Cigarettes per Day)						
10-19	0.46 (0.46, 0.46)	0.66 (0.66, 0.67)	0.84 (0.82, 0.85)	0.50 (0.50, 0.51)	1.00 (0.99, 1.01)	—
≥20	0.28 (0.28, 0.28)	0.47 (0.46, 0.47)	0.81 (0.79, 0.82)	0.33 (0.33, 0.34)	1.04 (1.03, 1.05)	—
Cigarette Smoking Frequency, 1st Trimester (Ref: 1-9 Cigarettes per Day)						
10-19	—	0.37 (0.37, 0.38)	0.57 (0.57, 0.58)	—	—	—
≥20	—	0.32 (0.32, 0.33)	0.49 (0.49, 0.50)	—	—	—
Cigarette Smoking Frequency, 2nd Trimester (Ref: 1-9 Cigarettes per Day)						
10-19	—	—	0.31 (0.30, 0.31)	—	—	—
≥20	—	—	0.30 (0.30, 0.31)	—	—	—
Cigarette Smoking Frequency, 1st Trimester (Ref: 0 Cigarettes per Day)						

© 2019 Soneji S et al. *JAMA Network Open*.

1-9	—	—	—	0.02 (0.02, 0.02)	1.37 (1.36, 1.38)	—
10-19	—	—	—	0.01 (0.01, 0.01)	1.41 (1.40, 1.42)	—
≥20	—	—	—	0.01 (0.01, 0.01)	1.54 (1.53, 1.56)	—
Cigarette Smoking Frequency, 2nd Trimester (Ref: 0 Cigarettes per Day)						
1-9	—	—	—	0.00 (0.00, 0.00)	1.35 (1.34, 1.36)	—
10-19	—	—	—	0.00 (0.00, 0.00)	1.38 (1.37, 1.39)	—
≥20	—	—	—	0.00 (0.00, 0.00)	1.56 (1.54, 1.58)	—
Cigarette Smoking Frequency, 3rd Trimester (Ref: 0 Cigarettes per Day)						
1-9	—	—	—	—	1.22 (1.21, 1.23)	—
10-19	—	—	—	—	1.27 (1.26, 1.28)	—
≥20	—	—	—	—	1.43 (1.41, 1.45)	—

Source: Authors' analysis of 2011-2017 U.S. Live Birth Certificate data.

© 2019 Soneji S et al. *JAMA Network Open*.

eFigure. Prevalence of Current Cigarette Smoking Among Females Aged 18-44 Years, Every Day Smoking Among Current Cigarette Smokers, and Quit Attempt Within Past Year Among Female Current Cigarette Smokers

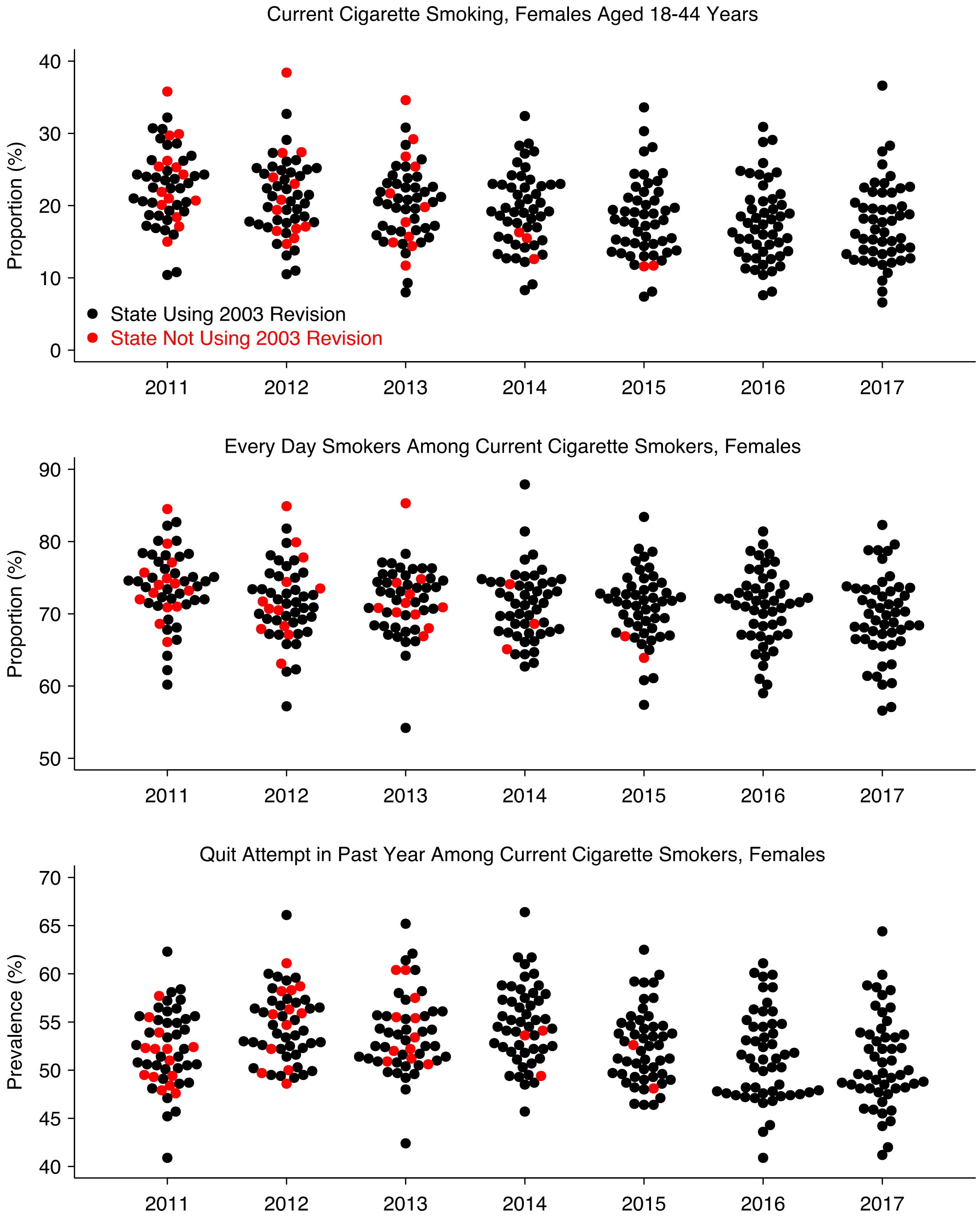

Source: Centers for Disease Control and Prevention, State Tobacco Activities Tracking and Evaluation (STATE) System. Based on 2011-2017 Behavioral Risk Factor Surveillance System data.

© 2019 Soneji S et al. *JAMA Network Open*.
